# The effect of triple reuptake inhibitor toludesvenlafaxine on neurological function in cerebral ischemic rats

**DOI:** 10.3389/fphar.2023.1073099

**Published:** 2023-04-21

**Authors:** Xiaohui Sun, Tian Wang, Lin Zhou, Ce Zhang, Fenghua Fu

**Affiliations:** School of Pharmacy, Key Laboratory of Molecular Pharmacology and Drug Evaluation (Yantai University), Ministry of Education, Collaborative Innovation Center of Advanced Drug Delivery System and Biotech Drugs in Universities of Shandong, Yantai University, Yantai, Shandong, China

**Keywords:** toludesvenlafaxine, triple reuptake inhibitor, antidepressants, cerebral ischemia, CREB pathway, neuroprotective effects

## Abstract

**Purpose:** The aim is to investigate the effect of toludesvenlafaxine (Tdv), a reuptake inhibitor of serotonin, norepinephrine, and dopamine, on the neurological function in cerebral ischemic rats and the underlying mechanisms.

**Material and Methods:** Middle cerebral artery occlusion/reperfusion (MCAO/R) model was induced in rats and the neuroprotective effects of Tdv were evaluated by infarct size, Garcia test, and beam walking test. Neuronal apoptosis in the peri-infarct area was observed by TUNEL staining. And the apoptosis-related proteins were evaluated with Western blotting. The role of CREB pathway in effect of Tdv was also investigated using Western blotting and immunofluorescence.

**Results:** In the MCAO/R model, administration of Tdv reduced the infarct size, promoted neural functional recovery, decreased the expression of Bax and Caspase-3, and increased the expression of Bcl-2 and BDNF. In addition, Tdv reduced neuronal apoptosis in the peri-infarct area. Tdv increased the expression of phosphorylated CREB. The application of the specific CREB inhibitor, compound 666-15, could reverse the anti-ischemic cerebral injury of Tdv in MCAO/R rats.

**Conclusion:** Tdv ameliorated cerebral ischemic injury through reducing neuronal apoptosis and increasing the expression of BDNF via the activation of CREB pathway.

## 1 Introduction

Stroke is a cerebrovascular disorder with high mortality rate and prevalence that occur worldwide including China. Ischemic stroke is commonly caused by the occlusion of the blood vessel, which is due to arterial thrombosis, and results in generating hypoxia condition in the brain. These episodes activate the cascade of injury, which causes apoptosis of brain cell (Zhao et al., 2017). In clinical therapy, management of cerebral ischemia can be achieved using of antiplatelet drugs and intravenous injection of recombinant tissue plasminogen activator (rt-PA). It has been reported that long term use of antiplatelet drug increases the risk of bleeding which leads to cerebral hemorrhage (Diener et al., 2004). The application of rt-PA was restricted by narrow therapeutic windows and high risk of bleeding transformation (Wang et al., 2018). Thus, in recent year, more emphasis was focused on the research related to alternative medicine for the management of ischemic stroke.

The death of brain cells after ischemic stroke is an important reason for neurological dysfunction. Although neuronal function is abnormal, the cells in ischemic penumbra are still viable. Therefore, the rescue of ischemic penumbra tissue is particularly critical in improving the prognosis of stroke (Li et al., 2021). The cAMP-response element binding protein (CREB) pathway regulates neurogenesis, neuronal survival, and synaptic plasticity after ischemic stroke (Chen et al., 2019). CREB kinases can phosphorylate CREB on the transcriptional regulatory site Ser133. Phosphorylated-CREB binds to cAMP response element (CRE) sites thereby induces the expression of nerve growth factor (NGF), brain-derived neurotrophic factor (BDNF), and other neurotrophic growth factors (Riccio et al., 2006). As an essential modulator of synaptic plasticity in the central nervous system, BDNF supports neuronal survival and promotes the growth and differentiation of new neurons, which exerts positive implications for stroke recovery (Bae et al., 2020). Furthermore, there are some researches showing that CREB attenuates neuronal apoptosis through regulating the expression of pro-survival and pro-apoptotic members of Bcl-2 family and caspase-3 (Hardingham et al., 2002). These findings demonstrate that CREB pathway has a neuroprotective effect by regulating the expression of BDNF and Bcl-2 after ischemic cerebral injury. 666-15, a CREB specific inhibitor, is used to evaluate the action of CREB in the apoptotic mechanism (Guan et al., 2019).

Existing evidences from clinical and animal experiments indicated that selective serotonin reuptake inhibitor (SSRI) and serotonin norepinephrine reuptake inhibitor (SNRI) improved the recovery of stroke patients by directly augmenting neuronal plasticity, neurogenesis, and neuronal differentiation (Liu et al., 2014). Fluoxetine, a SSRI anti-depressant drug, might provide a promising therapy in cerebral ischemia due to its neuroprotective effects. Fluoxetine shows a property of inhibiting neurons apoptosis and increasing neurorestorative effect (Khodanovich et al., 2018). Furthermore, pre-treatment with duloxetine, a SNRI, protected pyramidal neurons in the CA1 region from cerebral ischemia-reperfusion injury (Lee et al., 2016). Dopamine is a neurotransmitter that cannot be ignored in depression treatment. The role of dopamine in stroke recovery has also been studied. Dopamine was beneficial for the recovery of ischemic stroke, especially the recovery of motor function, learning and memory, and cognitive function (Kaushik et al., 2020). However, it has been suggested that sustained autoxidation of dopamine could lead to an excessive accumulation of toxic quinones and cytotoxic free radicals, which would be detrimental to stroke recovery (Lelos et al., 2012).

Toludesvenlafaxine (Tdv) is a new chemical entity and a potential triple reuptake inhibitor. Tdv increased levels of serotonin, norepinephrine, dopamine in synaptic cleft (Zhu et al., 2021). Since the protection of dopamine to against cerebral ischemia is controversial, it is unknown whether Tdv has beneficial effects in ischemic stroke. In addition, investigating the behavioral functional recovery and detailed molecular mechanisms of Tdv is necessary for guiding clinical medication. Nimodipine, a dihydropyridine calcium channel blocker, effectively inhibits transmembrane Ca^2+^ inward flow following cell depolarization. It has effects on multiple targets which are directly associated with brain injury and neurodegeneration. Thus ischemic stroke has been well-studied disease as a potential action for nimodipine (Carlson et al., 2020). Numerous studies have also shown that the neuroprotective effect of nimodipine was attributed to the inhibition of inflammation and oxidative stress, anti-apoptosis, and promotion of axonal sprouting and BDNF expression (Lin et al., 2019; Feng et al., 2020). Therefore, we used nimodipine as a positive control medicine.

## 2 Materials and methods

### 2.1 Animals

This study was performed according to the Institutional Guidelines for the Care and Use of Animals which was approved by the Yantai University. A total of 120 adult male Sprague–Dawley rats weighing 250–280 g were used in this experiment. Three rats died due to hemorrhage during Middle cerebral artery occlusion/reperfusion (MCAO/R), and others survived until following test, including 72 rats were used TTC staining, 24 for Western blotting and others were participated in immunofluorescence. All rats were kept in standard environment of temperature and humidity, and a 12 h light-dark cycle, with free access to food and water.

### 2.2 Drugs administration and grouping of animals

**FIGURE 1:**
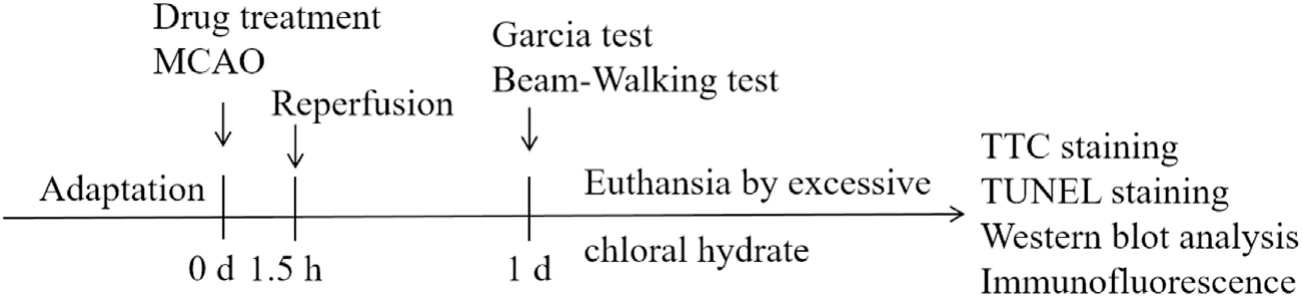
Schematic diagram of treatment, MCAO/R and behavioral testing.

Rats were randomly divided into the following groups: Sham, MCAO/R model, Nimodipine 8 mg/kg, Tdv 4 mg/kg, Tdv 8 mg/kg. Nimodipine and Tdv were administered by gavage at 30 min before MCAO. The experimental design is shown in Figure 1.

### 2.3 Transient middle cerebral artery occlusion

Transient focal cerebral ischemia was induced by MCAO in rat, as previously described (Ren et al., 2021). Rats were fasted for 12 h before the experiment and anesthetized with isoflurane. The neck of rats was sterilized by iodophor and the left middle cerebral artery was exposed. The middle cerebral artery was occluded by a thread plug. Reperfusion was performed by the withdrawal of the filament at 1.5 h of MCAO. The body temperature of animal was maintained at 36.5°C–37.5°C.

### 2.4 TTC staining and quantification of infarct size

The infarct size was evaluated using 2,3,5-triphenyl tetrazolium chloride (TTC) staining. TTC reacts with dehydrogenase in normal tissues. But dehydrogenase activity in ischemic tissues decreases and did not produce changes with it. Thus the non-infarcted part of the brain slice will be red and the other part will appear white. After 1.5 h of MCAO followed by 24-h reperfusion, rats were deeply anesthetized and sacrificed by overdose of chloral hydrate. The brain was removed rapidly and placed in −20°C for 20 min. Then the brain was sliced into 2 mm coronal sections and stained with 2% TTC for 20 min at 37°C. After the brain slices of the rats were taken by a digital camera, the infarct area was analyzed by an image processing system. The calculation of the area of the cerebral infarction (white stained) and the area of the non-infarct (red stained) were performed by an experimenter who was blinded to the groups.

### 2.5 Neurological deficits score

Garcia test was performed at 24 h after reperfusion by an experimenter who was blinded to the groups (Ren et al., 2021). Garcia test consisted of 6 tests including spontaneous activity, limb symmetry, forelimb walking, climbing, side stroking and tentacles touching. The neurological function was graded on a scale of 0–18. The higher score, the better neurological function.

### 2.6 Beam-walking test

The paw of rats with MCAO/R might fall or slip between the wires when walking on a grid (3 cm × 3 cm). If this occurred, it was recorded as a foot fault (Li et al., 2021). The total number of steps and the number of wrong steps within 3 min were recorded. The stagger rate was calculated with the following formula: the number of wrong steps/the total number of steps × 100%.

### 2.7 Western blotting

The total protein extracted from penumbra of rats (n = 3) were prepared according to the manufacturer’s instructions. The protein concentration in brain was determined by BCA kit. Aliquots containing 15 μL protein were separated by SDS/PAGE, transferred to a PVDF membrane and blocked with 5% non-fat dried milk in TBST for 4 h at 25°C. Membranes were then incubated (4°C overnight) with primary antibodies CREB (1 : 1,000), p-CREB (1 : 500), Bcl-2 (1 : 500), Bax (1 : 1,000), Caspase-3 (1 : 1,000), BDNF (1 : 1,000) or β-actin (1 : 1,000). Washed with TBST for 3 times, 10 min each. They were incubated with secondary antibody (1 : 1,000) for 1 h at 25°C, washed with TBST for 3 times with 10 min/time. The content of protein was determined by Image J and corrected the differences between samples by β-actin.

### 2.8 Immunofluorescence and confocal analysis

#### 2.8.1 TUNEL assay

The anesthetized-animals were transcendentally perfused with 0.9% saline and 10% buffered paraformaldehyde. Brains were removed from the skull immediately and post-fixed in 10% buffered paraformaldehyde at 4°C overnight. The fixed-brain was transferred to 30% or 15% sucrose solution for 12 h at 4°C. Sliced into 14 μm sections on a cryostat.

For double staining with TUNEL and neurons, sections were first stained with mouse anti-NeuN (1 : 500) antibody overnight at 4°C, then washed with PBST for 6 times and incubated with Alexa Fluor 488-conjugated secondary antibody (1:300) at room temperature for 2 h. After staining by neurons, sections were washed with PBST for 6 times, and then stained with TUNEL apoptosis assay Kit (Beyotime) according to the manufacturer’s instructions. The numbers of TUNEL^+^ cells in randomly selected image areas were counted. In addition, the data from three rats were used to participate in statistical analysis for each group.

#### 2.8.2 Localization of p-CREB and neurons

Cryosections for immunostaining was performed following the previously described method. In order to identify the localization of p-CREB and neurons, cerebral sections were permeabilized with 0.5% Triton X-100 in PBS for 30 min, blocked with 10% horse serum (PBS) for 1 h and then incubated in the primary antibodies, rabbit anti-p-CREB (1 : 500) and mouse anti-NeuN (1 : 500) antibody, overnight at 4°C. Then, washed with PBST for 6 times, cerebral sections were incubated with Cy5 and Alexa Fluor 488-conjugated secondary antibody (1 : 300) 37°C for 1 h. Washed with PBST for 6 times again and DAPI was added for 30 min. Immunofluorescence was visualized using a Laser Scanning Confocal Microscope (CLSM; ZEISS LSM 800, Germany).

### 2.9 Statistics

All data were presented as mean ± SD. Differences among multiple groups were assessed by one-way ANOVA followed by Tukey’s *post hoc* test. Behavioral tests were expressed as median with range and were analyzed by Krystal-Wallis test. The *p* < 0.05 were considered statistically significant.

## 3 Results

### 3.1 Toludesvenlafaxine alleviated neurological deficits and reduced cerebral infarction size

**FIGURE 2:**
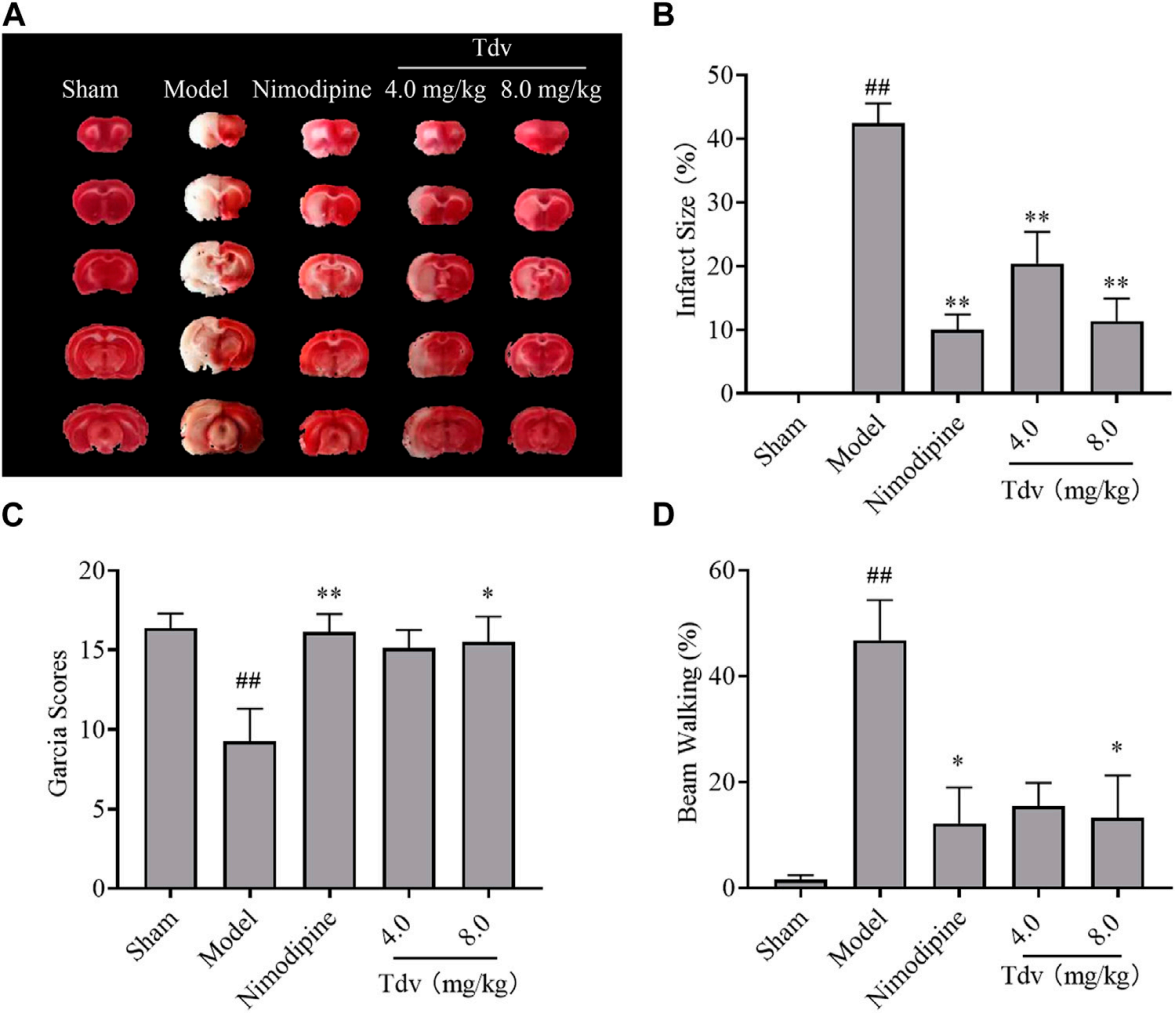
Toludesvenlafaxine alleviated neurological deficits and reduced cerebral infarction size. **(A)** TTC staining; **(B)** Cerebral infarct size; **(C)** Garcia Scores; **(D)** Beam-walking test. Data were expressed as mean ± SD and behavioral tests were expressed as medium ± range, n = 8. ^##^ *p* < 0.01 compared with sham group; ^*^ *p* < 0.05, ^**^ *p* < 0.01 compared with model group.

TTC staining showed that the brains of MCAO/R rats displayed obvious necrosis after ischemic injury. Compared with the model group, Tdv or Nimodipine treatment significantly reduced cerebral infarct size in MCAO/R rats (*p* < 0.01). Treatment with Tdv or Nimodipine significantly increased the Garcia scores and alleviated the beam-walking rate when compared with those of MCAO/R rats (*p* < 0.05, *p* < 0.01) (Figure 2 **)**. These results suggested that Tdv could improve neurological functions in MCAO/R rats, which was consistent with nimodipine. In addition, we found that Tdv 8.0 mg/kg showed the best neuroprotective effect.

### 3.2 Toludesvenlafaxine increased the expression of BDNF and decreased apoptosis

**FIGURE 3:**
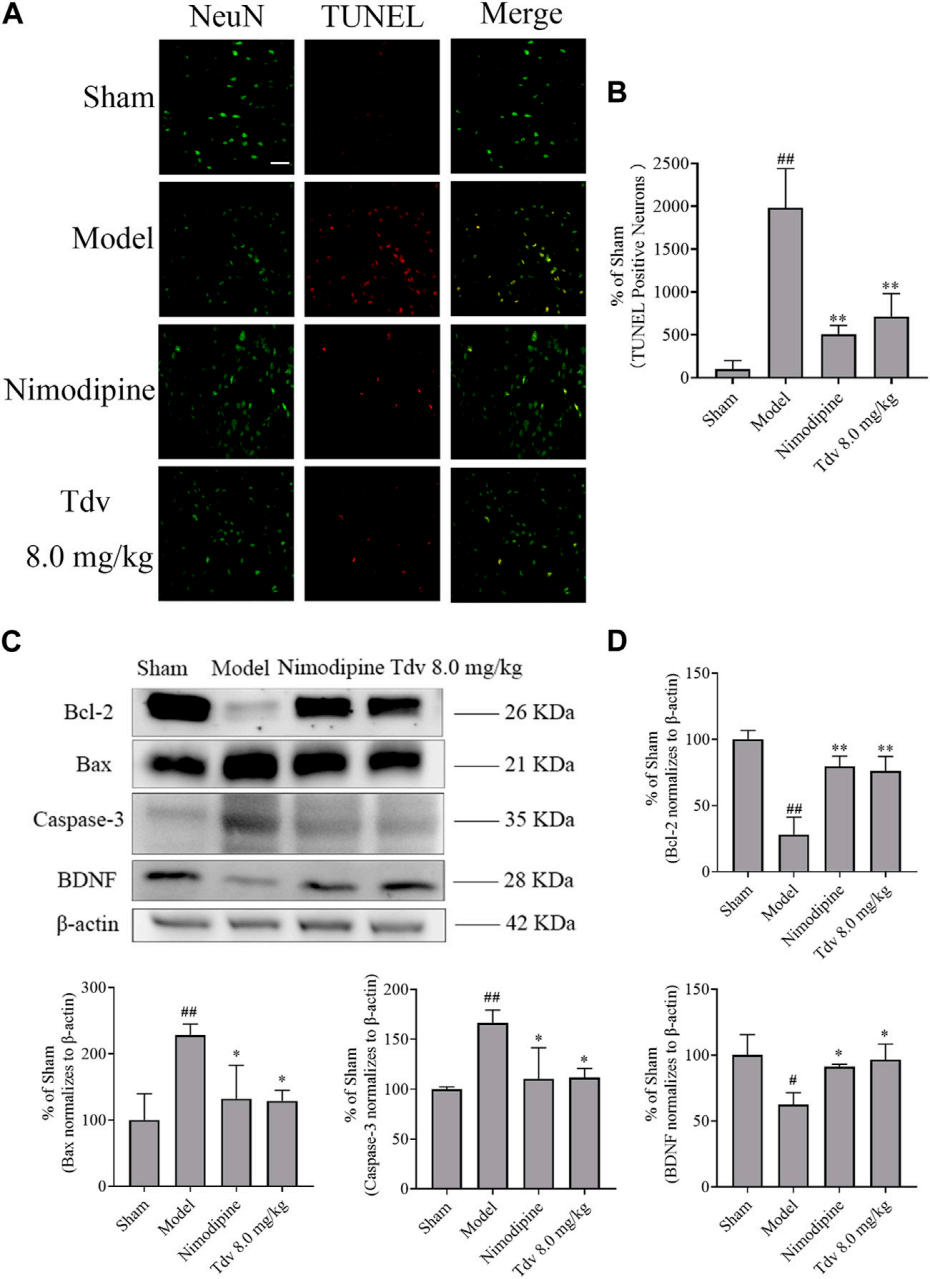
Toludesvenlafaxine increased the expression of BDNF and decreased apoptosis. **(A)** TUNEL staining; **(B)** The percentages of TUNEL positive cells (n = 3); **(C)** The expression of BDNF and anti-apoptotic protein; **(D)** Statistical results of Western blot. Immunofluorescence of data are mean ± SD, n = 3. ^##^ *p* < 0.01 *versus* sham, and ^**^ *p* < 0.01 *versus* model, scale bar = 50 μm. Western blot of data are mean ± SD, n = 3. ^#^ *p* < 0.05, ^##^ *p* < 0.01 *versus* sham, and ^*^ *p* < 0.05, ^**^ *p* < 0.01 *versus* model.

BDNF plays an important role in the neurological functional recovery in ischemic rat. We found that the expression of BDNF in the model group was significantly lower than that in the sham group (*p* < 0.05). Compared with model group, both Tdv (8.0 mg/kg) and Nimodipine upregulated the BDNF expression in cerebral ischemic rats (*p* < 0.05). The findings suggested that Tdv elevated the BDNF expression in rats with ischemic stroke (Figure 3).

A significant increase in apoptosis was observed in the brain of rats after MCAO/R (*p* < 0.01). What’s more, the expression of the caspase-3 and Bax significantly increased, and the Bcl-2 in cerebral cortex of penumbra was decreased after stroke (*p* < 0.05, *p* < 0.01). Compared to post-stroke, Tdv (8.0 mg/kg) reduced the number of apoptotic cells (*p* < 0.01). Furthermore, Tdv (8.0 mg/kg) increased the Bcl-2 and decreased the level of the caspase-3 and Bax (*p* < 0.05, *p* < 0.01). Like Tdv, Nimodipine also showed the same effect of increasing the expression of BDNF and decreasing apoptosis (*p* < 0.05, *p* < 0.01).

### 3.3 Toludesvenlafaxine induced the activation of the CREB pathway to promote functional recovery after stroke

**FIGURE 4:**
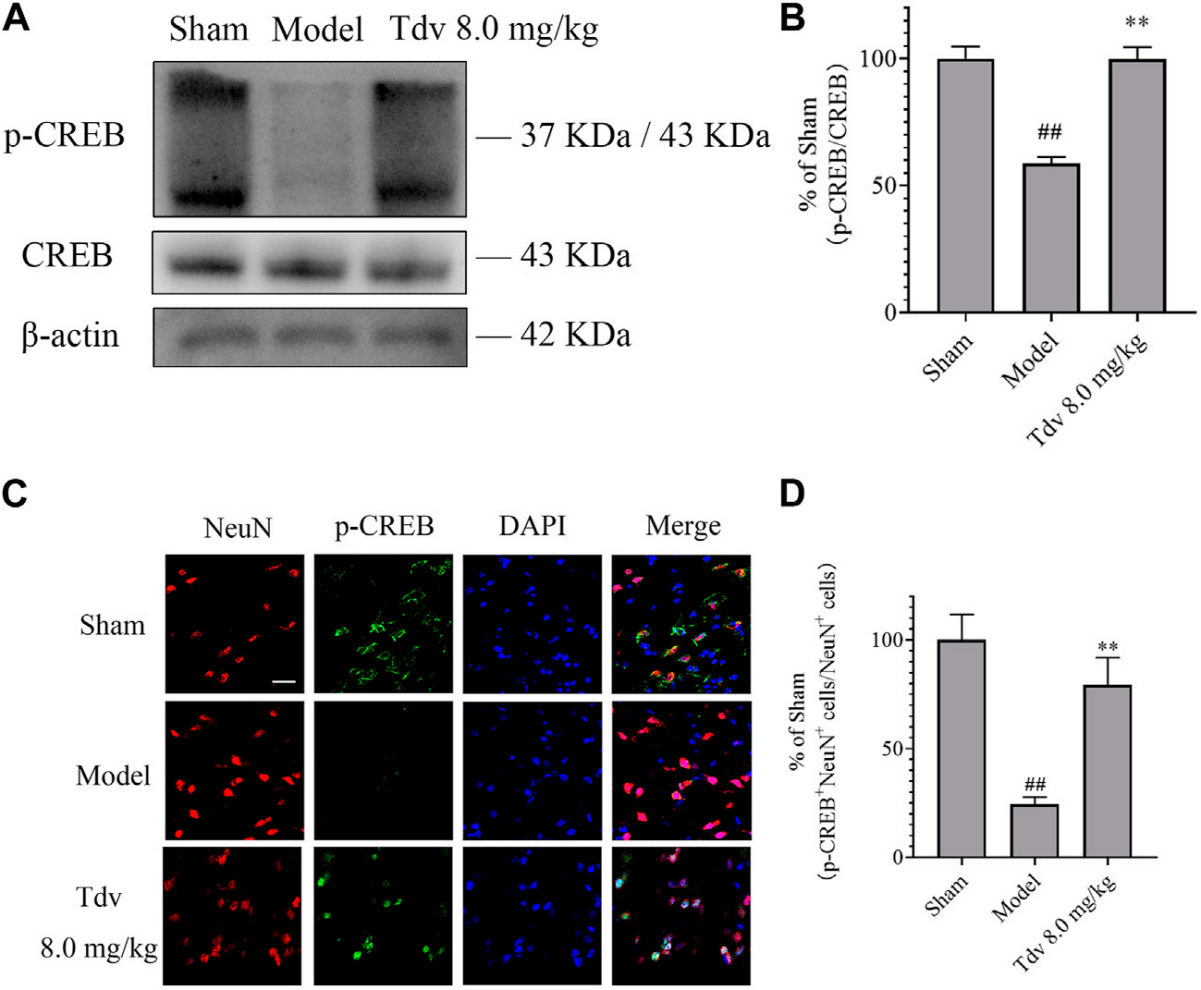
Toludesvenlafaxine induced the activation of the CREB pathway to promote the functional recovery after stroke. **(A)** The expression of p-CREB; **(B)** Statistical results of Western blot; **(C)** Immunofluorescence of p-CREB and NeuN, scale bar = 50 μm; **(D)** Statistical results of p-CREB^+^ cells. Western blot of data is mean ± SD, n = 3. ^##^ *p* < 0.01 *versus* sham, and ^**^ *p* < 0.01 *versus* model. p-CREB^+^ cells of data are also mean ± SD, n = 3. ^##^ *p* < 0.01 *versus* sham, and ^**^ *p* < 0.01 *versus* model.

The effect of Tdv on the expression of CREB and p-CREB in peri-infarct cerebral cortex was evaluated. Compared with sham group, the expression of the p-CREB of the rats in MCAO group markedly downregulated (*p* < 0.01). Tdv (8.0 mg/kg) elevated the level of the p-CREB when compared with model group (*p* < 0.01). Furthermore, double immunofluorescence staining indicated that p-CREB was expressed in cytoplasm and nucleus of neurons. The expression of p-CREB positive neurons was lower at 24 h after MCAO when compared to the sham group (*p* < 0.01). And the expression of p-CREB positive neurons was greater in Tdv group when compared to the model group (*p* < 0.01). Collectively, our data indicated that p-CREB was upregulated in neurons after Tdv treatment (Figure 4).

### 3.4 CREB inhibitor reversed the neuroprotective effects of toludesvenlafaxine

**FIGURE 5:**
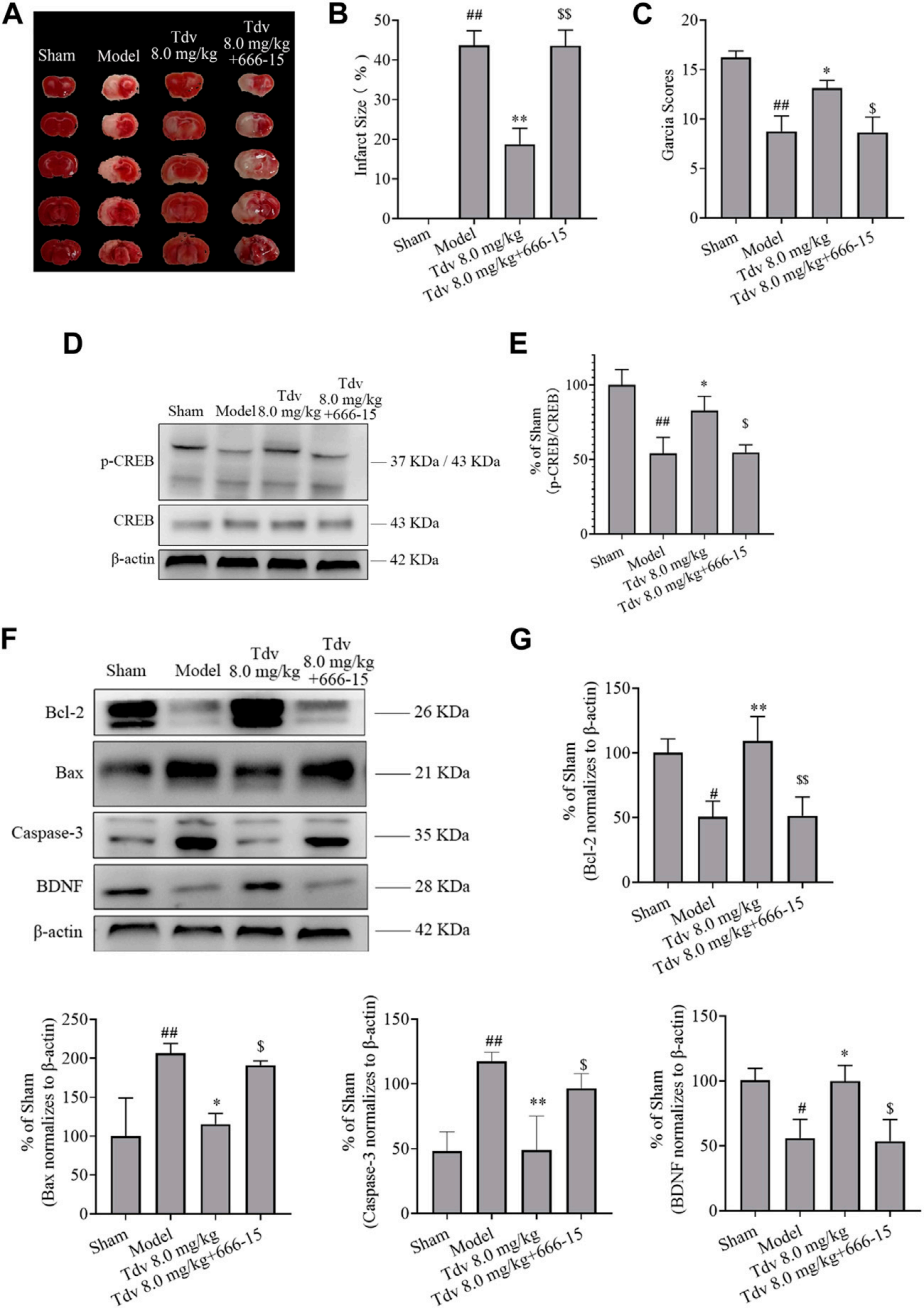
CREB inhibitor reversed the neuroprotective effects of toludesvenlafaxine. **(A)** TTC staining; **(B)** Cerebral infarct size (n = 8); **(C)** Garcia Scores (n = 8); **(D)** The expression of p-CREB; **(E)** Western blot results of p-CREB (n = 3); **(F)** The expression of BDNF and anti-apoptotic protein; **(G)** Western blot results of BDNF and anti-apoptotic protein (n = 3). ^#^ *p* < 0.05, ^##^ *p* < 0.01 *versus* sham; ^*^ *p* < 0.05, ^**^ *p* < 0.01 *versus* model; ^$^ *p* < 0.05, ^$$^ *p* < 0.01 *versus* Tdv.

Our results have proven that CREB-mediated sig+naling pathways are involved in the effect of Tdv on apoptosis and of BDNF in the progression of cerebral ischemia. To further prove this conclusion, we investigated the effects of 666-15 (CREB inhibitor) on the neuroprotective effects in the Tdv-treated (8.0 mg/kg) rats after MCAO/expression R. As shown in Figure 5, pretreatment with the CREB inhibitor 666-15 resulted in the inhibition of the neuroprotective effect of Tdv on the infarction size and neurological deficits (*p* < 0.05, *p* < 0.01). As shown in Figures 5D, E 666-15 almost completely reversed the expression of p-CREB, suggesting that the CREB pathway is associated with the cerebral protective effect of Tdv (*p* < 0.05, *p* < 0.01). In order to further analyze whether activation of CREB played a role in the attenuation of apoptosis and promotion of BDNF expression by Tdv, Western blotting were used. Interestingly, the results revealed that the effect of Tdv on apoptosis and BDNF expression were blocked upon inhibition of CREB pathway, which again suggested that the neuroprotective effects of Tdv was directly related to the upregulation of CREB signaling activities (*p* < 0.05, *p* < 0.01).

## 4 Discussion

Monoamine reuptake inhibitors including fluoxetine, citalopram, clomipramine, duloxetine, and venlafaxine showed protective effects against cerebral ischemia (Park et al., 2015). Monoamine neurotransmitters play an important role in the function of brain. The evidence demonstrated the changes of concentration of monoamine neurotransmitters observed in ischemic stroke (Matsumoto et al., 1984). Citalopram increasing 5-HT level in the dorsal raphe of rats improved the exercise recovery. And 5-HT_1A_ receptor antagonist reversed the anti-dyskinetic effects of SSRIs ( Conti et al., 2014 ). Norepinephrine has anti-inflammatory properties and is involved in neuronal plasticity in central nervous system. Atomoxetine, a selective norepinephrine reuptake inhibitor, increased the extracellular norepinephrine concentration in the rat brain and protected pyramidal neurons from ischemic insult (Park et al., 2015). It was reported that Tdv displayed high-affinity binding to SERT, NET and DAT and therefore inhibited the reuptake of serotonin, norepinephrine, and dopamine. In addition, microdialysis study showed that Tdv increased the levels of serotonin, norepinephrine, and dopamine in central nervous system after oral administration, and exhibited significant antidepressant effects in animal models (Zhu et al., 2021). Hence, we speculate that Tdv not only has antidepressant properties, but may also have a positive effect on cerebral ischemia. Interestly, the results of our study demonstrated the neuroprotective of Tdv in the rat MCAO/R model. Tdv treatment reduced infarct size. And Tdv at dose of 8.0 mg/kg had the most prominent effect on the reduction of infarct size. Many studies showed that dopamine plays a contradictory role in attenuating cerebral ischemic brain injury. On the one hand, cerebral ischemia results in a massive release of dopamine and glutamate in the striatum, which induces depletion of ATP level and leads to neuronal cells loss (Kim et al., 2013). The release of dopamine exerts neurotoxic effects after ischemia, which directly contributes to the cells death and nigrostriatal lesions (Oliva et al., 2013). However, dopamine D2 receptor agonists such as pergolide, bromocriptine, and lisuride showed neuroprotective effects after ischemic stroke. Dopamine has also been reported to play an important role in modulating neuronal plasticity in cerebral ischemia (Momosaki et al., 2017). Our experimental results have shown that the elevation of serotonin, norepinephrine, and dopamine in the central nervous system may display neuroprotective effect after ischemic stroke. In other words, Tdv showed a protective effect on cerebral ischemia.

Tdv had a potential to ameliorate cerebral ischemia/reperfusion injury. Tdv treatment improved outcomes after cerebral ischemia. Garcia test included spontaneous activity, symmetry in the movement of four limbs, fore-paw outstretching, climbing, body proprioception, and vibrissae touch. The scores decreased obviously after cerebral ischemia. And administration of Tdv increased the scores of Garcia test. Furthermore, motor impairments of ischemic stroke rats were evaluated in the present study using the beam-walking test. The beam-walking rate of rats with cerebral ischemia increased and Tdv administration resulted in a downward trend, which indicated that Tdv can improve the neurological function after cerebral ischemia.

Cerebral ischemia leads to pathological injury in the brain and the inhibition of apoptosis may be an ideal option to salvage the neurons of the penumbra. Apoptosis is controlled by pro-apoptotic family members and anti-apoptotic family members (Fricker et al., 2018). Bcl-2, an anti-apoptotic protein, promotes the survival of neuronal cells through regulating permeabilization of the mitochondrial outer membrane. Bax, a pro-apoptotic protein, translocates into the mitochondria and induces neuronal apoptosis. Caspase-3, a member of the endoproteases family, also regulates apoptosis signaling networks (Wang et al., 2021). We found that rats of cerebral ischemia displayed an upregulation of neuronal apoptosis and expression of pro-apoptotic proteins in the cerebral cortex. Tdv reduced the infarct size and the number of apoptotic neurons in the cerebral cortex. Further findings demonstrated that Tdv prevented neuronal apoptosis by down-regulating expression of Bax, caspase-3 and up-regulating expression of Bcl-2.

BDNF, a neurotrophic factor, is widely distributed in the brain, especially in hippocampus and cortex. BDNF has neurogenesis, neuroprotection, and angiogenesis effects. Furthermore, upregulation of BDNF has a neuroprotective effect after experimental stroke (Liu et al., 2022). Similarly, our study found a decreased expression of BDNF in the cortex of the ischemic penumbra. Tdv increased BDNF in the cortex of the ischemic penumbra, which suggested that Tdv attenuated cerebral injury by promoting BDNF expression.

CREB is involved in neural stem cell proliferation, neuronal differentiation, learning and memory, and other physiological and pathological activities (Lee et al., 2004). CREB signaling pathway exerts a prominent effect on cerebral ischemia - reperfusion injury. In addition, CREB will be activated in the ischemic penumbra area in a model of focal cerebral ischemia and inhibition of CREB phosphorylation has an adverse effect in reducing brain injury (Liu et al., 2022). The suppression of CREB pathway inhibited the expression of BDNF and Bcl-2, which affected neuronal survival after cerebral ischemia (Du et al., 2010). The present study proved that Tdv increased the expression of p-CREB. In order to further validate the role of CREB pathway in protecting the brain after cerebral ischemia, 666-15, a specific CREB inhibitor, is used in the present study. CREB inhibitor reversed the increase of expression of BDNF, Bcl-2 and the decrease of expression of Bax, caspase-3 caused by Tdv administration. Therefore, it is reasonable to speculate that Tdv exhibited neuroprotective effects by regulating the CREB signaling pathway.

Nimodipine has neuroprotective effects on many disorders of brain, such as subarachnoid hemorrhage, ischemic stroke, traumatic brain injury, and migraine (Kumar et al., 2022). Here, we used nimodipine as a positive control to observe the neuroprotective potential of Tdv. Both nimodipine and Tdv demonstrated a reduction in cerebral infarct size and improvement in neurological impairment following cerebral ischemia, which was associated with inhibition of neuronal apoptosis and promotion of BDNF expression. There were no any significant differences observed in two groups. Although in cerebral ischemia, Tdv did not show a more pronounced advantage over nimodipine, Tdv itself has an antidepressant effect (Zhu et al., 2021), which would be more applicable to the treatment of post-stroke depressed patients. Additionally, Tdv may be safer for patients with depressant who were facing a high risk of ischemic stroke at the same time.

In conclusion, our results suggested Tdv, a triple reuptake inhibitor, exhibited neuroprotective effects on cerebral ischemic injury and ameliorated the neurological deficits via regulating the CREB signaling pathway. Tdv might provide a promising therapeutic strategy in the management of patients with ischemic stroke.

## Data Availability

The original contributions presented in the study are included in the article/supplementary materials, further inquiries can be directed to the corresponding author.
